# Endoscopic band ligation with hypertonic glucose cushion in the treatment of ileal pouch prolapse

**DOI:** 10.1093/gastro/goaa089

**Published:** 2020-12-07

**Authors:** Bo Shen

**Affiliations:** Center for Inflammatory Bowel Disease, Columbia University Irving Medical Center-New York Presbyterian Hospital, New York, NY, USA

## Introduction

Restorative proctocolectomy with ileal pouch–anal anastomosis (IPAA) is the surgical treatment of choice of patients with ulcerative colitis (UC) or familial adenomatous polyposis who require the removal of the colon. While this reconstructive surgery significantly improves patients’ quality of life, post-operative structural, inflammatory, and functional complications are common. In our Pouch Center, we have noticed a growing number of patients presenting with dyschezia, feeling of incomplete evacuation, and frequent bowel movements. Clinical, endoscopic, and radiographic evaluation of cohorts of patients has identified a cluster of disease phenotypes that are collectively diagnosed with floppy pouch complex (FPC) [1]. Common forms of FPC are pouch prolapse, afferent limb syndrome, and pouch folding. In a survey of 269 surgeons, 35 indicated that they had been taking care of a total of 83 patients with symptoms suggestive of pouch prolapse, such as external prolapse of tissue, sense of obstructed defecation, seepage, and pain [2]. In an early study of 3,176 patients who underwent ileal pouch surgery, 11 were diagnosed with pouch prolapse (0.3%) [3]. The true prevalence may be higher in clinical practice. Pouch prolapse may be mucosal or full-thickness; and anterior, posterior, or circumferential. The treatment of pouch prolapse has traditionally been medical (such as suppositories and creams) and surgical (such as pouch pexy, mesh placement, and excision) [1, 2, 4]. Over the past decade, we have developed a novel endoscopic approach for the treatment of pouch prolapse.

## Case report

A 48-year-old female patient developed worsening daily dyschezia with excessive straining, a sensation of incomplete evacuation, frequent bowel movement, daytime or nocturnal seepage, and perianal discomfort since staged laparoscopic restorative proctocolectomy and IPAA for refractory UC 5 years prior. She suffered from early satiety, bloating, and weight loss of 15 lbs from her healthy baseline. She had no fever or night sweat. She was diagnosed as having pouchitis and was treated with antibiotics. Her diarrhea and bloating symptoms partially responded to antibiotic therapy. However, her symptoms almost became antibiotic-dependent. Her past medical history included hypothyroidism. She had three vaginal deliveries before colectomy and 10 years ago underwent hysterectomy for uterine fibroid bleeding. Physical examination showed well-healed skin scars at the stoma site and suprapubic area. There was evidence of perianal dermatitis. She was able to expel the balloon in office-based anopouch manometry. The manometry showed no paradoxical contractions. Barium defecography showed anterior pouchocele and partial obstruction of the pouch outlet. Magnetic resonance imaging 1 year prior showed no fistula, presacral sinus, or abscess.

**Figure 1.**
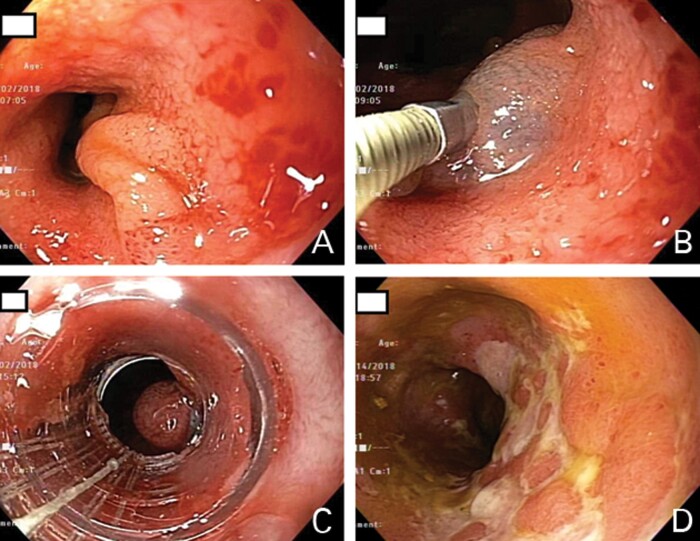
Endoscopic treatment of pouch mucosal prolapse. (A) Distal anterior pouch mucosal prolapse, partially blocking the outlet; (B) submucosal injection of hypertonic glucose into the prolapsed area; (C) placement of endoscopic bands; (D) follow-up endoscopy showing resolution of the mucosal prolapse. The endoscopically treated area was covered with mucosal scars.

The diagnosis of pouch prolapse was suspected. After obtaining diagnostic and therapeutic information, we performed an elective outpatient pouchoscopy with the patient under conscious sedation. The patient was put in a left lateral position. The absence of the cuff was consistent with the patient’s history of IPAA with mucosectomy and hand-sewn anastomosis. The mucosa and lumen of the afferent limb, pouch inlet, and tip of the ‘J’ were normal. However, mucosal prolapse at the anterior wall of the distal pouch was found, almost blocking 80% of the lumen of the anal canal during endoscopic suction (**Figure 1A**). We performed a submucosal injection of 6 mL of 50% glucose to lift the prolapsed mucosa (**Figure 1B**), followed by the endoscopic deployment of five bands (Speedband Superview Super 7™ Multiple Band Ligator Boston Scientific, Marlborough, MA) (**Figure 1C**). The procedure took a total of 20 minutes and was not eventful. The patient tolerated the procedure well and was discharged home after observation for 30 minutes in the endoscopic recovery room. During the first 2 days after the procedure, the patient was able to expel the bands and experienced mild anal discomfort likely from the pinch of the contracting bands. The patient gradually noticed an improvement in symptoms and eventually had a resolution of dyschezia and sensation of incomplete evacuation and decreased bowel frequency. A follow-up pouchoscopy was performed that showed resolution of the mucosal prolapse. The prolapsed area was replaced with ulcers from the band-ligation procedure. The anterior wall of the treated distal pouch was felt to be stiffer (**Figure 1D**). The patient has been followed for 1.5 years to date and remains symptom- and antibiotic-free.

## Discussion

Pouch prolapse along with other phenotypes of FPC that cause significant morbidities and compromise the quality of life is increasingly being recognized. The etiology and pathogenesis are largely unknown. The diagnosis of pouch prolapse is based on clinical, endoscopic, radiographic, and histologic evaluation. Common presentations include dyschezia, the sensation of incomplete evacuation, frequent bowel movements, perianal discomfort, and bloating. Patients may have a partial response to antibiotic therapy, probably thanks to the component of small-intestinal bacterial overgrowth. Pouch prolapse may coexist with functional dyssynergic defecation or structural complications of the pouch, such as anterior cuffitis and pouch–vaginal fistula. The prolapsed portion of the pouch can easily be detected by positioning the tip of the endoscopy at the anal canal and suction. The classic endoscopic feature of pouch prolapse is a protruding bowel wall under suction, typically at the anterior wall, completely or partially blocking the pouch outlet. Prolapse of the distal anterior pouch body or anterior cuff may be associated with endoscopic inflammation in the area. Barium defecography plays an important role in the diagnosis of pouch prolapse and other forms of FPC. On an X-ray, pouch prolapse often presents with distal pouchocele and outlet obstruction. Histologic evaluation often shows classic features of prolapse, such as elongated mucosal crypts and intramucosal fibrosis.

The management of pouch prolapse can be challenging. Patients with concurrent pouch prolapse and distal pouchitis or cuffitis may respond to therapy with topical agents, such as mesalamine, corticosteroids, or valium. Patients with concurrent paradoxical contractions on manometry may benefit from pelvic-floor biofeedback therapy. Digital vaginal pushing during defecation may also help. Patients refractory to the conservative therapy have traditionally required surgical intervention. The surgical modalities for pouch prolapse include pouch pexy, mesh placement, and mucosal excision [3–5]. The outcome of surgical treatment has setbacks. We noticed that post-operative recurrence has been common. Three of 11 patients with pouch prolapse who were surgically treated developed J-pouch failure requiring conversion to continent ileostomies [3].

Endoscopic treatment of prolapsed bowel has been explored. Small prolapse can be endoscopically resected with hot snares [6]. Endoscopic band ligation was performed for this patient with an optimal outcome. In fact, surgical band ligation was performed for the treatment of rectal prolapse [7], in a similar fashion to the treatment of hemorrhoids [8]. However, endoscopic treatment using a variceal banding device for pouch prolapse is novel. In addition, submucosal injection of hypertonic glucose before band ligation can minimize the risk of bleeding and the risk of the accidental trapping of the posterior wall of the vagina into the banding cap causing vaginal fistula. In fact, hypertonic glucose has been used to treat varices [9], hemorrhoids [10], and pleural effusion [11], presumably due to its sclerosing effect. The combined effects of mechanical banding and chemical sclerosing from hypertonic glucose made the anterior pouch wall stiff and resistant to prolapse.

This case demonstrates that the efficacy and safety of combined endoscopic banding and submucosal injection of hypertonic glucose for pouch prolapse. We anticipate that there will be a growing number of patients with pouch prolapse in clinical practice and the novel endoscopic approach may be considered as one of the first-line therapies.

## Funding

None.

## Conflicts of interest

None declared.
